# Protein Oxidative Damage in UV-Related Skin Cancer and Dysplastic Lesions Contributes to Neoplastic Promotion and Progression

**DOI:** 10.3390/cancers12010110

**Published:** 2020-01-01

**Authors:** Antonella Tramutola, Susanna Falcucci, Umberto Brocco, Francesca Triani, Chiara Lanzillotta, Michele Donati, Chiara Panetta, Fabiola Luzi, Federica Iavarone, Federica Vincenzoni, Massimo Castagnola, Marzia Perluigi, Fabio Di Domenico, Federico De Marco

**Affiliations:** 1 Department of Biochemical Sciences, Sapienza Rome University, P. le Aldo Moro 5, 00185 Rome, Italy; antonella.tramutola@uniroma1.it (A.T.); francesca.triani@uniroma1.it (F.T.); Chiara.lanzillotta@uniroma1.it (C.L.); marzia.perluigi@uniroma1.it (M.P.); 2 Department of RiDAIT, the Regina Elena National Cancer Institute IRCCS, via Elio Chianesi 53, 00144 Rome, Italy; susanna.falcucci@gmail.com (S.F.); umberto.brocco@gmail.com (U.B.); 3 Department of Pathology, University Campus Biomedico, via Alvaro del Portillo 21, 00128 Rome, Italy; m.donati@unicampus.it; 4 UOSD Dermopathology, the San Gallicano National Dermatological Institute IRCCS, via Elio Chianesi 53, 00144 Rome, Italy; Chiara.panetta@ifo.gov.it; 5 UOSD Dermatological and Reconstructive Plastic Surgery, the San Gallicano National Dermatological Institute IRCCS, via Elio Chianesi 53, 00144 Rome, Italy; fabiolaluzi@yahoo.it; 6 Istituto di Biochimica e Biochimica Clinica, Università Cattolica, Dip. di Diagnostica di Laboratorio e Malattie Infettive, Fondazione Policlinico Universitario A. Gemelli, IRCCS, 00168 Rome, Italy; federica.iavarone@unicatt.it (F.I.); Federica.vincenzoni@unicatt.it (F.V.); massimo.castagnola@unicatt.it (M.C.)

**Keywords:** ultraviolet, solar radiation, skin cancer, carcinogenesis, cancer promotion, protein oxidation, redox proteomics, protein damage, stress response

## Abstract

The ultraviolet (UV) component of solar radiation is the major driving force of skin carcinogenesis. Most of studies on UV carcinogenesis actually focus on DNA damage while their proteome-damaging ability and its contribution to skin carcinogenesis have remained largely underexplored. A redox proteomic analysis of oxidized proteins in solar-induced neoplastic skin lesion and perilesional areas has been conducted showing that the protein oxidative burden mostly concerns a selected number of proteins participating to a defined set of functions, namely: chaperoning and stress response; protein folding/refolding and protein quality control; proteasomal function; DNA damage repair; protein- and vesicle-trafficking; cell architecture, adhesion/extra-cellular matrix (ECM) interaction; proliferation/oncosuppression; apoptosis/survival, all of them ultimately concurring either to structural damage repair or to damage detoxication and stress response. In peri-neoplastic areas the oxidative alterations are conducive to the persistence of genetic alterations, dysfunctional apoptosis surveillance, and a disrupted extracellular environment, thus creating the condition for transformant clones to establish, expand and progress. A comparatively lower burden of oxidative damage is observed in neoplastic areas. Such a finding can reflect an adaptive selection of best fitting clones to the sharply pro-oxidant neoplastic environment. In this context the DNA damage response appears severely perturbed, thus sustaining an increased genomic instability and an accelerated rate of neoplastic evolution. In conclusion UV radiation, in addition to being a cancer-initiating agent, can act, through protein oxidation, as a cancer-promoting agent and as an inducer of genomic instability concurring with the neoplastic progression of established lesions.

## 1. Introduction

Non-melanoma skin cancer (NMSC), because of its comparatively low invasion and metastatic activity, is commonly considered a rather benign condition. As a matter of fact, despite a range of therapeutic options and the recent developments in non-invasive or minimally invasive diagnostic procedures, recurrences and systemic spread are not negligible events even for those patients with negative histological margins and negative local lymph-nodes. In addition, because of the prevalent topographic localization of lesions (head and neck, shoulders and décolleté region), their removal is per se a demanding surgical task, a reason for worry and anxiety for the patients because of the possibility of a disfiguring outcome and a major issue for national health systems all over the world.

A large amount of research work has been dedicated to the molecular mechanisms of ultraviolet (UV) carcinogenesis with most of studies focusing on mechanisms and effects of genetic damages. Extensive data have been collected, the occurrence of genetic damage as both direct and indirect consequence of UV radiation is conclusively demonstrated and unanimously accepted, the vast range of biochemical adducts to DNA have been described in detail and their relative carcinogenic potential has been clearly established [1] Meanwhile, the potential contribution of UV-dependent protein damage to skin carcinogenesis remains largely underexplored, despite protein damage being a major driving force of carcinogenesis. Indeed, the prevention of cancer critically relies on the efficient coordinated actions of numerous proteins for appropriate DNA damage repair (DDR) as is strikingly emphasized by the case of Xeroderma Pigmentosum (XP). In this condition, inactivating mutations of genes encoding the NER proteins generate dysfunctional proteins causing a severe impairment of DNA repairing mechanisms and the consequent sharply increased risk of cancer development, mostly occurring in sun exposed areas [2]. In XP patients the UV radiation generates the same amount of DNA damage as in normal patients; however, because of inadequate protein function, the genetic lesions persist longer, increasing the chance for unrepaired DNA to undergo replication. This generates mutations at an increased pace ultimately leading to cancer development. In the same line are the data about the p53 status whose functional alterations have a most powerful oncogenic activity so that they are largely perceived as a universal driving force of carcinogenesis. In sun-exposed skin, UV-dependent p53 mutations are highly frequent [3]. These mutations occur decades before the onset of neoplastic lesions, are associated with a decrease or lack of p53 function impairing the DNA repair activity [4] and the sunburn-dependent apoptosis [5]. The UVB DNA lesions are, therefore, replicated and transmitted to descendant cells and clonal expansion takes place paving the way to dysplastic evolution and cancer development. The crucial role of efficient removal of DNA damage, crucial in the outcome of radiation damage is further supported by a number of observations in radio-resistant species (briefly and elegantly reviewed in [6]) as well as by human ex vivo data [7]. These authors report that in melanoma patients, upon UV irradiation the same amount of cyclobutane pyrimidine dimer (CPD) and 6-4 photoproducts (6-4PP) DNA lesions are generated as in control patients, and that DNA repair occurs with the same kinetics in both cases. This indirectly supports the idea that persistency (inversely related to proteins repairing efficiency) rather than generation of DNA damage is the crucial step in carcinogenesis. Nonetheless, direct evidence about the impact of UV in human proteome structure and function and their potential role in carcinogenesis is scant and difficult to gather.

In previous works we reported about the pattern of carbonylation induced by UV-B radiation in HPV16 transformed and in normal human keratinocytes and suggested that dysfunctional proteins might result in cell homeostasis impairment and the promotion of cellular degeneration [8,9]. Later, in a redox proteomic study on cervical neoplastic and pre-neoplastic lesions, we showed that elevated levels of carbonylation are associated with pre-neoplastic lesions and that carbonylation mostly affected proteins involved in cell morphogenesis and terminal differentiation and suggested that their deregulated function is a driving force in neoplastic progression [10]. Conversely, in fully neoplastic tissues, a comparatively improved control of oxidative damage seemed to occur as shown by the selective reduction of carbonyl adducts on key detoxifying/pro-survival proteins [10]. As a whole these data underscore, although in another biological system, the critical role played by the proteome structural and functional status in cell homeostasis and cancer progression.

Wu CL et al. [11] reported about protein expression and Redox-dependent changes found in UV-B treated human diploid fibroblast showing that a number of proteins involved in protein folding and DNA synthesis were upregulated, and that thiol oxidation mostly occurred on proteins involved in cytoskeleton, metabolism and signal transduction. Gueranger and colleagues in an in vitro study [6] found that UVA, in the presence of the photosensitizing agent 6-Thioguanine, induces a sharp burden of protein carbonylation and protein thiol oxidation. This was specifically confirmed to occur on the Ku, OGG-1; MYH and RPA proteins, all members of the DNA repair proteome, and was associated with a markedly impaired NER activity. Lately, McAdam et al. has demonstrated in an in vitro study that protein damage inhibits DDR and determines mutation risk [12].

To contribute to the comprehension of proteins oxidative damage to UV and solar carcinogenesis, here we report about the proteome oxidative damage found in vivo in human solar-induced advanced skin neoplastic lesions.

## 2. Results

**Figure 1:**
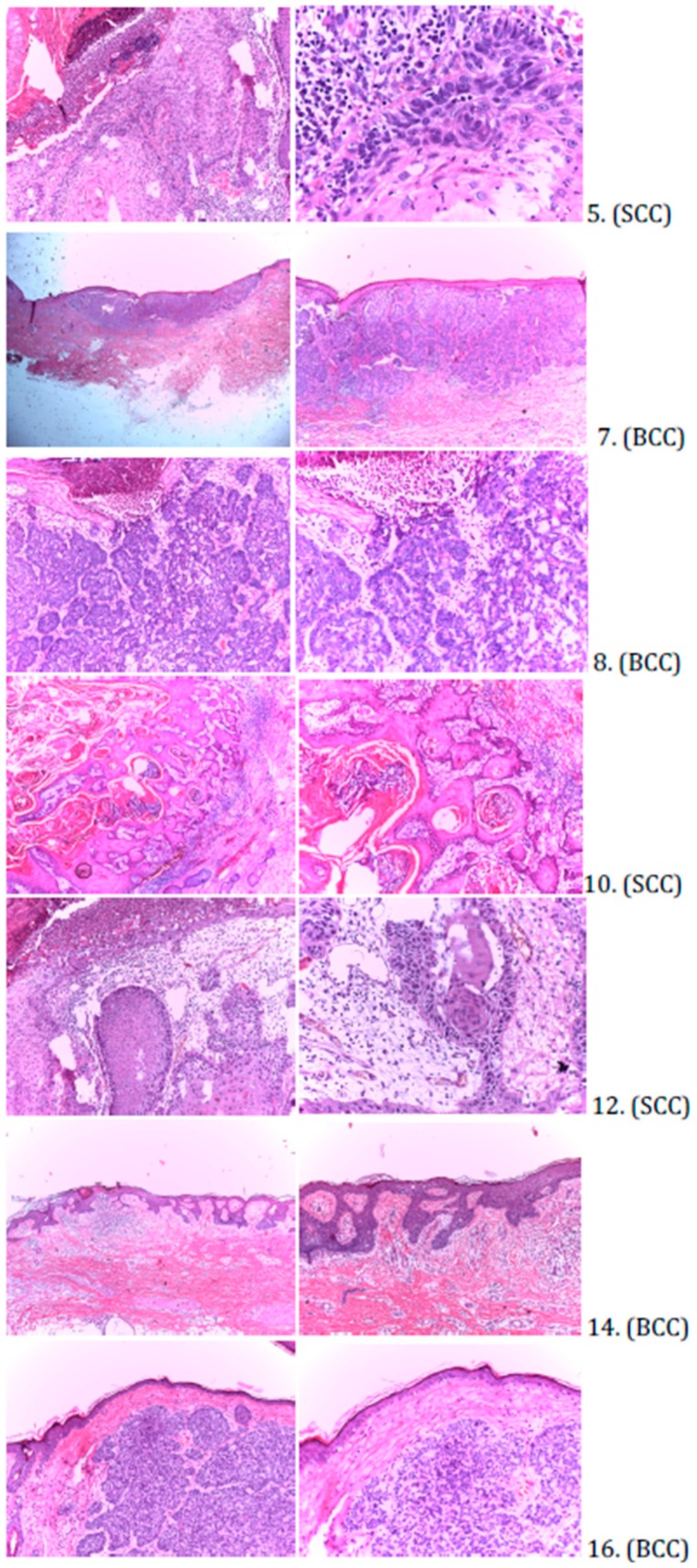
Histopathological presentation tissue samples and pathological diagnosis (SCC: squamous cell carcinoma) (BCC: basal-like cell carcinoma). Low magnification pictures on the left hand side (Pts 5; 8; 10; 12 and 16, 4× magnification; pts. 7 and 14 magnification 1.5×). On right hand side particulars at higher magnification (pts 5 and 12, 20×; pts 7 and 14, 4×; Pts 8, 10 and 16, 10×).

The resident ethical committee had preliminarily and thoroughly approved this work. Seventeen patients, among those fulfilling the inclusions criteria and giving their consent, were included in the study. Solar lesions are pathological conditions evolving through a lifelong process. To gain sound data about the very late state of proteome oxidation it is necessary to keep to a minimum the contribution of other concurring/confounding factors such as the p53 status and the possible infection with human papillomavirus (HPV). Patients’ clinical presentation, histopathological diagnoses, p53 and HPV status are reported in Table 1 and their histopathological sections are depicted in Figure 1.

### 2.1. P53 Status

**Figure 2:**
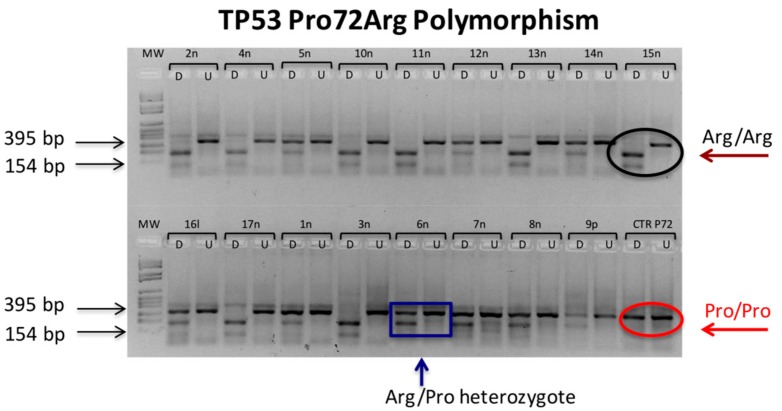
*BstUI* restriction fragment length polymorphism (RFLP) analysis of p53 codon 72 dimorphism. The presence of a guanosine at nucleotide 216 in the exon 4th amplicon can be revealed *BstUI* restriction. Thus, the presence of two 131 and 208 bp bands in digested lane (D) stands for the R72 variant (black circle) whereas the full length 339 bp undigested band indicates the P72 variant (red circle). Both digested and undigested bands in D lanes indicate Pro/Arg heterozygous (blue box). (U) Undigested DNA.

The p53 protein is a master anti oncogenic protein and the major coordinator of DNA damage repair and oxidative stress response following UV exposure [13]. A most frequent polymorphism has been described for p53 at the codon 72 involving a single nucleotide exchange (c > g) generating either the proline (P72) or the arginine (R72) variant. Such a polymorphism occurs in a proline-rich region involved in the apoptosis control [14] and generates two alleles that are biologically distinguishable. P72 is a stronger inducer of p21, one of p53 effectors involved in cell cycle checkpoint [15] and the K72 allele has a higher ability in localizing to mitochondria and inducing apoptosis [16]. R72 is also more susceptible to HPV E6 degradation [17] and is associated with an increased risk of Uteri cervix cancer [18]. Both abilities, the p21 inducing and the apoptosis inducing one, are critical to the cellular response to UV damage although no clear indications are presently available about their potential role in the evolution of solar-induced lesions. The codon 72 status of our patients, evaluated by restriction fragment length polymorphism (RFLP) [19], is shown in Figure 2.

As can be seen, 8/17 patients were heterozygous for the Pro/Arg genotype, 9/17 were homozygous for the Arg/Arg genotype and none of them was Pro/Pro homozygous. The analysis of single point mutation by an amplification refractory mutation system polymerase chain reaction (ARMS-PCR) [20,21], although conceptually elegant proved labour intensive, highly delicate and poorly reproducible and did not yield consistent results (data not shown). The UV radiation, through the generation of the cyclobutane pyrimidine dimers (CPDs) and the pyrimidine (6-4) pyrimidone photoproducts (6-4PPs) at di-pyrimidine dimers, specifically promotes the TT > CC or the CT > CC transversion [1]. These mutations are considered a hallmark of UV DNA damage and their occurrence at specific sites in the exon 7 of the p53 gene has been proposed as a lifelong cumulative index of UV exposure [22]. The sequence analysis of p53 exon 7 revealed a large number of minor alterations occurring in the Exon 7 either on the UV-related hot-spot codons 245 and 247/248 or in other positions. In no case, however, were the detected lesions the so-called UV-B signature indicating that the TT > CC or CT > CC transversion, although specific for UV-B lesions, is a comparatively infrequent occurrence.

### 2.2. Human Papillomavirus (HPV) Transformation

HPVs are direct powerful oncogenic agents with high epitheliotropic specificity. Both Alpha HPVs (comprising the types HPV 6; HPV 11; HPV 16; HPV 18; HPV 31; HPV 33; HPV 35; HPV 56; HPV 58) and Beta HPVs (including HPV 5; HPV 8; HPV 19; HPV 20; HPV 21; HPV 22; HPV 23; HPV 24; HPV 38; HPV 75; HPV 80; HPV 92 types) are well known modulators of tissue response to UV radiation [9,23] and to cancer related oxidative stress (OS) [10]. The alpha HPVs have a distinct cutaneous mucosal anogenital tropism, however they can also be found in normal and pathological skin. Their continuous expression is necessary for the induction and maintenance of the neoplastic state which is achieved through the disruption/modulation of a number of functions including the response to UV radiation [9]. This viral activity, irrelevant in cervical carcinogenesis, can be of biological significance in HPV bearing skin lesions. In addition, HPV16 expression in an ex vivo study, proved able to generate a specific signature on the cell redox-proteome [10]. Interestingly, OS in addition to be an elective trait of the neoplastic phenotype, is also a major specific effect of UV radiation, although generated through an indirect mechanism. Conversely, the cutaneous beta HPVs have a sharp cutaneous tropism and, being able to potentiate the UV genotoxic damage, are critical co-factors to UV radiation in the early steps of skin carcinogenesis while they are not necessary once the neoplastic phenotype is established, rather acting with a “hit and run” mechanism, as indicated by their progressively decaying prevalence with increasingly severe dysplastic and neoplastic lesions [24,25]. Thus both genera, if present, have the potentials to significantly interact with the oxidative pattern of solar lesions even though this takes place with different mechanisms. As reported in Table 1 and Table 2, samples tested positive for Alpha HPVs and 1 tested positive for Beta HPV. However, none of those samples stained positive for the presence of the E4 protein, thus indicating that HPV genomes were not transcriptionally active.

### 2.3. Protein Oxidation

**Figure 3:**
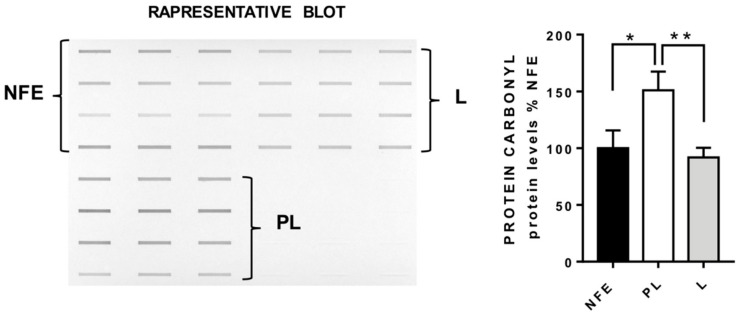
Slot blot analysis of total protein carbonylation. Panel **A** (left hand side): Slot blot of a representative sample from non-photo exposed (NPE), perilesional (PL) and lesional (L) groups. A triplicate of 4 samples per group is showed. Panel **B** (right hand side): Densitometric analysis of total protein carbonylation in NPE, PL and L groups. NPE is set as 100%. The bars show the averages of 7 samples per group ± standard error of the mean (SEM); * *p* < 0.05, ** *p* < 0.01.

Carbonylation is the most abundant protein oxidative adduct generated by UV radiation. Here, the total carbonylation of proteins from samples belonging to the three groups of comparison were evaluated by slot blot analysis as a surrogate index for total protein oxidation. As can be seen from Figure 3, the global protein carbonylation load in the perilesional (PL) areas was consistently more severe than in non-photo exposed (NPE) areas. Unexpectedly, the global carbonylation level was consistently higher in PL than in lesional areas (L), which presented oxidation values similar to that of NPE.

### 2.4. Redox Proteomics

**Figure 4:**
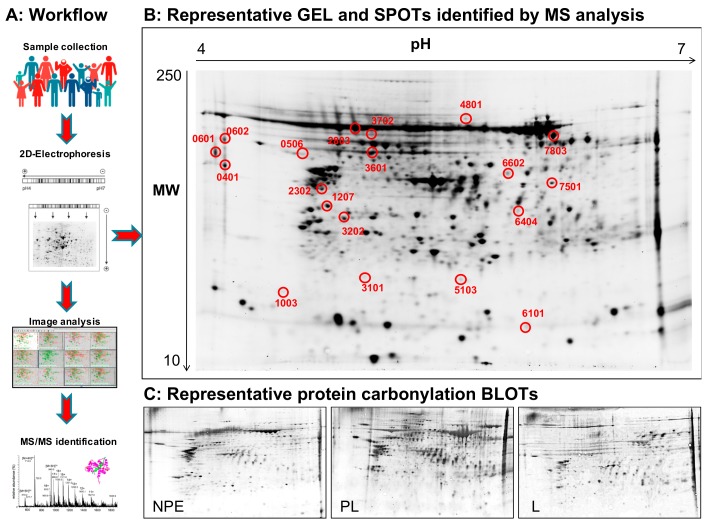
Two-dimensional (2D) electrophoresis images. Panel **A**: Workflow of redox proteomics approach employed in the study. Panel **B**: Representative 2D gel from NPE samples showing position and ID number of the spots identified to be differentially carbonylated in both PL vs. NPE and PL vs. L comparison groups. Proteins matching ID spots numbers are reported in Table 2. Panel **C**: Representative 2D blots from NPE, PL and L samples showing the different amount of total protein carbonylation.

To further investigate the effects of the different grade of oxidation observed by slot blot in the three groups of samples we ran a redox proteomics analysis for the identification of specifically carbonylated proteins (see workflow in Figure 4A). A set of 7 samples for each set was analysed comparing the L area, the corresponding PL area and the homologous NPE skin region among each other, establishing three groups of comparison PL vs. NPE, PL vs. L and L vs. NPE. One representative gel that display the number and the position of identified spots (and three representative blots, one for each set of samples, are shown in Figure 4B,C. As is evident by the images and in line with total carbonylation data, the comparison between of L and NPE groups did not show significant oxidation differences and did not yield significant data concerning the altered carbonylation of specific proteins (Figure 4C). Instead, the redox proteomic analysis of the other two groups of comparison (PL vs. NPE, PL vs. L) demonstrated the increased carbonylation of a total of 20 spots identified as 33 proteins with differential oxidation among one or more groups (Figure 4C and Table 2). The discrepancy between spots number and proteins number is due to the presence of multiple identification within the same spot, which is a common phenomenon in gel-based proteomics approaches. Due to the high number of redox-proteomic identified proteins these are listed in Table 2, together with their major determinants reported by mass spectrometry (MS) analysis: Uniprot ID, full name and acronym, corresponding gene acronym, identification score, peptide coverage, molecular weight (MW) and calculated isoelectric point (pI). Interestingly, the PL group demonstrated the altered carbonylation of 13 spots (24 proteins) when compared with the NPE group and of 10 spots (14 proteins) when compared with the L group. Intriguingly, of the 13 spots identified aberrantly oxidized in the comparison PL vs., NPE, 11 spots (20 proteins) resulted with increased oxidation, while 2 spots (4 proteins) presented decreased oxidation. Instead, of the 10 spots aberrantly oxidized in the comparison PL vs. L, spots 7 (9 proteins) resulted in increased oxidation, while 3 spots (5 proteins) presented decreased oxidation (Table 2). The evaluation of the number of proteins identified by redox proteomics approach and their fold of oxidation between the two sets of comparison confirms slot blot data posing the PL group as highly targeted by the formation of potentially toxic carbonyl groups, potentially toxic to cells. On the contrary the neoplastic L groups confirm, surprisingly, the reduction of protein oxidative damage suggesting the activation of protective mechanism to counteract the toxic effects on skin tissue.

In Table 3, a subset of proteins identified by MS analysis are grouped according to their major functions. As it can be seen, all listed proteins do actually cluster to a surprisingly narrow range of functional groups, namely: chaperoning and stress response, protein folding/degradation; apoptosis/survival; cell architecture, adhesion and motility; DNA damage repair; Moreover, all the above-named functional groups converge to two main physiological functions i.e.: cell damage response, repair and/or detoxication and cell survival/death.

### 2.5. STRING Analysis of Protein Networks

**Figure 5:**
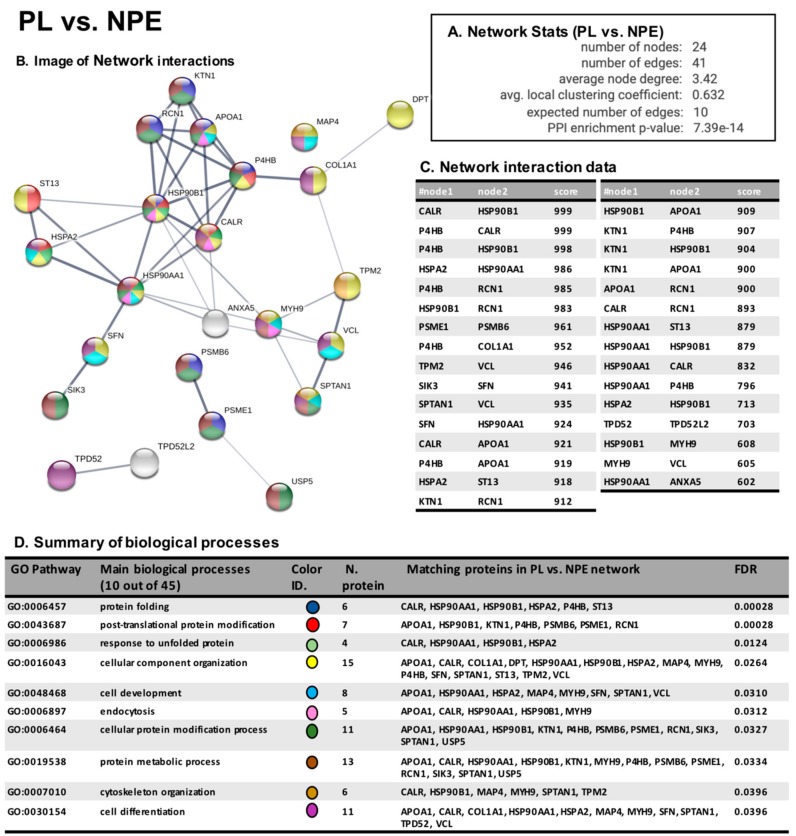
STRING analysis PL vs. NPE. Panel **A**: Network statistics reporting data concerning the number of nodes and edges, the average node degree, the average local clustering coefficient, the expected number of edges and the PPI enrichment *p*-value. Panel **B**: Network interaction image showing nodes and edges between the proteins identified. The thickness of the line indicates the strength of the interaction between the proteins. The colours of the sphere indicate the biological processes to which the protein belongs. Panel **C**: Network interaction table reports all the significant interactions (min 0.4) between the protein of the PL vs. NPE network. Panel **D**: Biological process table reports the main pathways to which the protein of the networks belongs. For each biological process identified, the corresponding Gene Ontology (GO) pathway, the number and identity of proteins and the false discovery rate is reported.

By using STRING software 11.0 [26,27], we performed the analysis of protein interaction network and of biological processes for the protein belonging to the two groups of analysis, PL vs. NPE and PL vs. L. This kind of bio-informatic analysis allows us to understand the biological implications of the aberrant carbonylation occurring on MS/MS-identified proteins. The analysis of the network generated by proteins aberrantly carbonylated in the comparison between PL and NPE (Figure 5A) showed a number of edges (41) higher than the expected (10) supporting that the carbonylated proteins have an increased number of interactions among themselves than would be expected for a random set of proteins of similar size, drawn from the genome. Protein interaction network is then reported as image and table (Figure 5B,C), and it indicates that the proteins are at least partially biologically connected, as a group. In table D of Figure 5 the main biological processes regarding the oxidized proteins belonging to the network generated by the comparison between PL and NPE are reported.

As expected, protein oxidation in PL samples concerns components of protein folding process, of unfolded protein response, of cell structure/organization and of cell development differentiation, suggesting that the impairment of these mechanisms might promote cancer progression.

**Figure 6:**
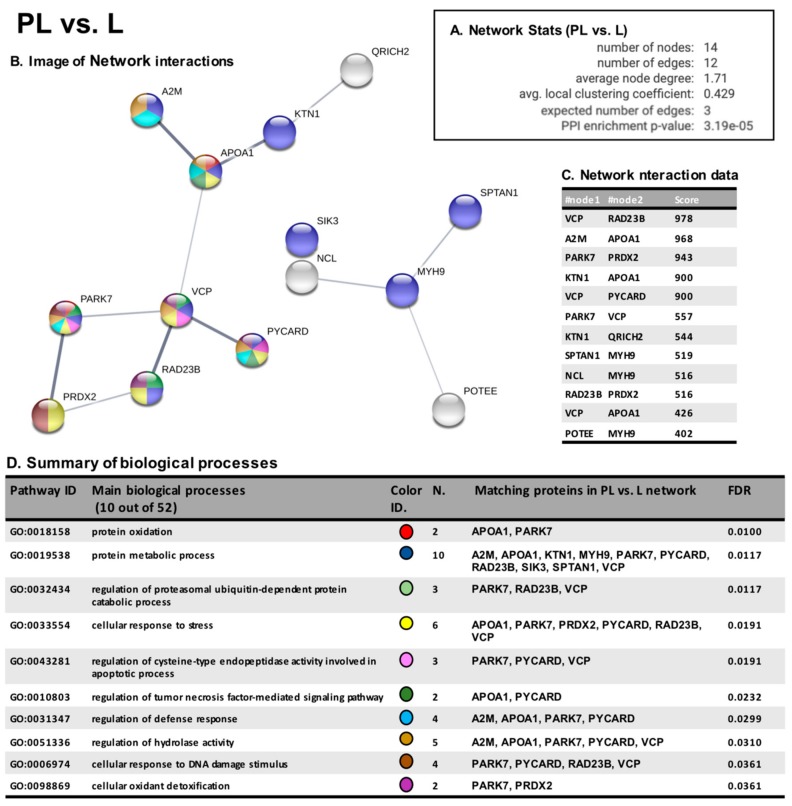
STRING analysis PL vs. L. Panel **A**: Network statistics reporting data concerning the number of nodes and edges, the average node degree, the average local clustering coefficient, the expected number of edges and the PPI enrichment *p*-value. Panel **B**: Network interaction image showing nodes and edges between the proteins identified. The thickness of the line indicates the strength of the interaction between the proteins. The colours of the sphere indicate the biological processes to which the protein belongs. Panel **C**: Network interaction table reporting all the significant interactions (min 0.4) between the protein of the PL vs. NPE network. Panel **D**: Biological process table reporting the main pathways to which the protein of the networks belongs. For each biological process identified, the corresponding GO pathway, the number and identity of proteins and the false discovery rate is reported.

About the comparison of PL vs. L areas, we observe a number of edges 4-times higher (12) than expected (4) (Figure 5A) supporting also in this case that most of the proteins are biologically connected, as is also demonstrated in the network image and interaction table (Figure 6B,C).

Intriguingly, table D of Figure 5, which reports the main biological processes involving the increasingly oxidized protein, shows the potential perturbation of mechanism involved in protein oxidation, response to stress conditions, regulation of degradative systems, response to DNA damage and OS detoxification, suggesting that the modulation of these pathways may aid neoplasia development and adaptation.

## 3. Discussion

A great number of studies have investigated the biological impact of ultraviolet radiation (UVR). So far, the vast majority of works have been focusing on the DNA damage while the well-known protein oxidative damage [28,29] remained largely underexplored. In recent years however, the potential contribution of protein oxidation to photo-aging and carcinogenesis has gained attention [30] and a number of in vitro studies have been published. Here we report about the protein carbonyl modifications found in vivo in solar-induced skin lesions and perilesional areas. Considering the lifelong natural history of solar lesions for a stringent interpretation of oxidative data, it is necessary to consider the possible contribution of two other relevant factors such as the p53 and HPV status. A dimorphism of the *TP53* gene at codon 72 has been described generating two biologically distinguishable variants, the P72 and the R72 variant, both critical to the cellular response to UV damage response. Addressing the still unresolved question of their respective implications in skin carcinogenesis is out of the scope of this communication, nonetheless it is interesting to note that the almost even prevalence of the Arg/Arg and Pro/Arg genotypes and the absence of the Pro/Pro genotype in our cases agrees with the data already reported for European populations [31] suggesting that, as far as the codon 72 is concerned, our series of patients do not represent a peculiar subset of population. Regarding the UV signature at specific hotspots in exon 7, the ARMS-PCR did not yield conclusive results while direct sequencing revealed a high number of point mutation, none of which however consisted in the canonical TT>CC or CT > CC transversion [1], suggesting that perhaps this kind of mutations, although specific, are comparatively infrequent in late lesions. Regarding HPV infection, alpha HPVs, and most frequently HPV16, this is sometimes found in dysplastic and neoplastic skin lesions where, if transcriptionally active, it may have a distinct impact on the oxidative status [9,10]. Conversely beta HPVs act as co-factors in the early steps of UV carcinogenesis potentiating and extending the persistence of DNA damage [24,25]. Their function is not necessary in late stages as shown by their decreasing prevalence in increasingly severe lesions. In both cases the continuous presence of viral oncogenes is required for their biological effects. In this work the tiny amount of available material did not permit an extensive viral characterization, however the lack of E4 protein expression indicates that none of the most prevalent beta HPVs were functionally active in our samples. Taken together the data about p53 and HPV status indicate that the series of samples here analysed includes no gross biasing factors and consequently the reported pattern of proteome oxidation can reasonably be attributed to the specific effect of solar radiation. The comparison of proteome carbonylation between the three groups under analysis, NPE, PL and L, clearly demonstrates a strong correlation of protein oxidative damage to the early steps of carcinogenesis, while a sort of cell adaptation to stress condition is observed in fully neoplastic cells.

### 3.1. Protein Carbonylation Critically Affects Protein Homeostasis

Within this context, the comparison of the oxidized proteome from PL versus the NPE areas, here considered as bona fide normal areas, identified the increased carbonylation of a number of proteins crucially involved in protein homeostasis (proteostasis) with specific roles in damage repair or degradation. These proteins are protein disulphide isomerase A1 (PDI-A1), heat-shock protein 90 (HSP90), glucose-related protein 94 (GRP94) also known as heat shock protein 90 member 1, heat shock-related 70 kDa protein 2 (HSPA2), calreticulin (Calr) and proteasome subunit beta type-6 (Psmb6). Most of these proteins act as molecular chaperones in the cell, thus representing the first line of defence against protein misfolding and aggregation. PDI catalyses the formation (the oxidation)/breakage (the reduction) of disulphide bonds, thus enabling their dynamic rearrangement [32] taking part in the processes of vesicles and particle endocytosis [33], cell/extra-cellular matrix (ECM) interaction [32] and forming/rearranging disulphide bonds of nascent proteins. In condition of persistent OS, PDI isomerase activity is strongly decreased leading to accumulation of misfolded proteins and activation of the unfolded proteins response (UPR) and Endoplasmic Reticulum (ER) stress response, which trigger apoptotic signalling in cancer cells [34,35,36]. The increased carbonylation of PDI-A1 was already reported to occur in in vitro irradiated human primary keratinocytes [8] and Fibroblasts [11]. Thus, present data, while confirming that PDI-A1 is a specific target for UVB induced OS, indicate that its carbonylated form actually occurs in solar lesions in vivo and might contribute to cell damage and cell transformation. The three members of the heat-shock proteins family HSP90, GRP94 and HSPA2 appear also to be severely carbonylated in perilesional areas. HSP90 specifically promotes TGFbeta signalling [37], a central pathway in skin homeostasis, and participates in cell differentiation by chaperoning cIAP1 [38]. Calr represents in the ER one of the main proteins with chaperone function, contributing with Calnexin to the PQC system of endoplasmic proteins. Furthermore, Calr, together with PDI is a constituent of the protein-loading complex (PLC) [39], thus directly controlling the activity of major histocompatibility complex (MHC) recognition and of the subsequent immune response [40]. At final, we observed the carbonylation of Psmb6 a component of the 20S core proteasome complex involved in the proteolytic degradation of most intracellular proteins including a subset of MHC-I presented antigen peptides. The increased carbonylation of all the above outlined proteins is consistent with their functional alteration as already reported by others and us [10,11,30,41,42]. Thus, we can assume that multiple crucial components controlling the structural/functional proteome integrity are thereafter severely impaired in PL areas respect to NPE tissue. Such alterations may result in improper folding of nascent proteins and inadequate refolding of stressed proteins, inefficient stabilization of the correctly folded conformations, accumulation of carbonylated dysfunctional/dysregulated proteins, all contributing to the high level of protein oxidative damage observed in PL.

### 3.2. Protein Carbonylation is an Independent Generator of DNA Damage

The accumulation of unfolded and over carbonylated proteins can concur to carcinogenesis through many possible mechanisms. However, two of them have already been proved and well detailed. First, oxidatively damaged proteins, acting as photo-damage sensitizers, through secondary reactive oxygen species (ROS) generation, have a distinct genotoxic activity and significantly contribute to the global UV-related genomic damage [43]. Second, oxidatively damaged proteins, in addition to the proven oxidation of key proteins specifically involved in DDR [6], per se induce a specific impairment of the DNA repairing mechanisms [12,41]. Therefore, in addition to the direct immediate UV genotoxicity, the oxidative proteomic damage is an independent generator of genetic damage and a distinct inducer of DDR impairment. As a consequence, genetic alterations tend to persist longer, increasing the chance to generate oncogenic mutations. Furthermore, misfolded/dysfunctional proteins can accumulate and precipitate in cell compartments and organelles generating inappropriate or deleterious effects even on pathways non-immediately hit by direct radiation or ROS-generated damage.

### 3.3. Protein Carbonylation Dysregulates Apoptosis Response

All these deleterious conditions, within a physiological context, would promptly lead to apoptosis activation eliminating cells with the potential to originate neoplastic clones. However, this protective mechanism is unlikely to be available within the photo-damaged tissue as a combined consequence of the UV-related p53 alterations [4] and of the increased carbonylation of multiple components of the apoptotic response. Remarkably, PDI-A1, HSPA2 and GRP94 beyond their role in proteostasis maintenance, have distinct apoptosis regulatory functions. PDI-A1 is an essential p53 activator following the UPR response [44], HSPA2 inhibits c-Jun, p38 and ERK activation by MAPK kinases [45]; suppresses the induction of senescence independently of p53 [46,47], and stabilizes the lysosome with cell survival effect [48] while the GRP94, regulates the ER stress-related suppression of apoptosis [49]. Moreover, two other key apoptotic determinants, Ubiquitin carboxyl-terminal hydrolase 5 (UCH-5) and Annexin-5 (Anx-5), appear to be carbonylated in PL compared to NPE areas. UCH-5 cleaves linear and branched multi-ubiquitin polymers and regulate the level of Lys-48-linked poly-ubiquitin tails, which compete with ubiquitinated p53/TP53 for proteasomal recognition and degradation. Thus, changes in UCH-5 levels have the potentials to represent a ROS operated mechanism of p53 regulation [50]. Anx-5 takes part in signal transduction and in negative apoptosis regulation and plays important roles in a number of neoplastic conditions [51]. Moreover, it takes relevant roles in cell–ECM interaction and in cell adhesion and motility [52] and has been proposed as a potential tumour marker [53].

### 3.4. Proteins Carbonylation Disrupt the Cell/Extra-Cellular Matrix (ECM) Interaction

A further key finding observed in PL areas compared to NPE areas is represented by the carbonylation of proteins involved in the cellular/ECM interactions, namely dermatopontin (Dpt) and Vinculin (Vcl). Dpt, an abundant component of non-collagenous ECM, mediate keratinocytes adhesion by cell surface integrin binding [54] and serves as a communication link between the fibroblast cell surface and ECM environment. It is expressed at a lower level in fibroblasts from patients with invasive tumours [55], indicating that a severely dysregulated Dpt activity is somewhat connected with deregulated growth. Vcl, a crucial member of focal adhesion and *zonula adherens,* is also considered an important factor in stress and mechano-torsional transduction [56]. Overall our data about Dpt and Vcl carbonylation, in addition to those on PDI-A1, HSP90, GRP94, Calr and Psmb6, which present differential roles in cell/ECM interaction [33,57,58,59], support that the complex network of cell/ECM communications is severely perturbed in PL areas, contributing to a tissue-specific inefficient containment of the dysplastic cells [60].

### 3.5. Proteome Carbonylation Promotes the Generation and the Progression of Transformant Clones

Overall, data generated by the comparison between PL and NPE support a role in cancer progression for the aberrant alteration of components of pathway involved in protein folding and degradation and in the regulation of proteostasis. As a consequence, DNA damage and proteome misfunction tend to persist longer increasing the chance for damaged DNA to undergo inappropriate repair or replication. Pro-carcinogenic mutations are, therefore, generated at an increased pace while cells bearing potentially carcinogenic alterations, inefficiently cleared because of the concomitant apoptosis dysregulation, can establish and grow. Moreover cell/matrix signalling, inflammation and immune reaction, are also critically subverted, unleashing transformant clones to expand and progress to an increasingly severe phenotype.

### 3.6. Antioxidant and Stress Response Pathways are Under-Oxidised in Neoplastic Areas

The subsequent analysis of the data generated by the comparison of PL vs. L aids to describe the molecular mechanisms by which the skin tissue responds to UVB irradiation promoting the proliferation of neoplastic clones. In L areas, we observed the apparently paradoxical condition of a reduced level of protein oxidation in neoplastic tissue as compared with the peri-neoplastic one. Nonetheless, these data are supported by the lack of significant identifications in the comparison between L and NPE, which sustain a decrease of protein oxidation in L areas compared to PL areas to levels similar to NPE regions. Consistently, similar findings have been reported about the cervical cancer vs. high-grade cervical dysplastic lesions [10] and an increased antioxidant response has been reported to account for an increased cell resistance to genotoxic and oxidative distress in a subset of Lung adenocarcinomas [61] and the hypothesis has been proposed that an adaptive selection of best fitting clones to the sharply pro-oxidant neoplastic environment takes places along with tumour progression. However, extreme caution is required in interpreting the reduced carbonylation level of a set of proteins within the context of the extensively deranged metabolisms of neoplastic cells. Furthermore, the actual pathological role of each of them is to be specifically assessed by purposely designed studies to come. Among the proteins identified with decreased carbonylation levels in L compared to PL areas a crucial role in tumour progression can be played by nucleolin (Ncl), protein/nucleic acid deglycase DJ-1 (DJ-1), peroxiredoxin 2 (Prdx-2) and transitional endoplasmic reticulum ATPase (Vcp), all belonging to antioxidant and stress response pathways. Overall, their reduced oxidation supports their conserved functionality and their contribution to an increased tolerance to the high level of OS in L areas. Indeed, DJ-1 has a far-ranging role in OS control and cell protection by reducing the level H_2_O_2_ [62], acting as a redox-sensitive chaperone and protease [63,64]), regulating mitochondrial morphology and mitophagy and modulating translation through a redox sensitive binding/releasing mechanism [63,65,66,67]. Prdx-2 plays a role in cell protection against oxidative stress by detoxifying peroxides and by acting as sensor of hydrogen peroxide-mediated signalling events. [68,69]. Vcp is involved in the formation of the transitional endoplasmic reticulum (tER). Moreover, through the recruitment of TP53BP1 at the DNA damage sites Vcp interconnects trafficking and organelle maintenance to the DDR [70,71]. The reduced carbonylation of these detoxifying players, in addition to the lack of oxidation of proteostasis components, as observed in PL areas, suggests that neoplastic cells may evolve protective mechanisms to survive and adapt to sharply pro-oxidant environment.

### 3.7. Protein Carbonylation Promote Genomic Instability in Neoplastic Areas

In contrast, L areas demonstrates the increased carbonylation of two proteins, the UV excision repair protein RAD23B (HR23B) and Ncl, that are involved in DNA stability and damage repair. HR23B, initially identified as a factor involved in initiation of global genome nucleotide excision repair (GG-NER) [72] where it acts as component of the *Xeroderma Pigmentosum* complex (XPC) that stabilizes and protect XPC complex from proteasomal degradation. The XPC:HR23B dimer is sufficient to initiate NER in response to cisplatin and UV-damaged double-stranded DNA damage [73]. Finally, HR23B interacts with 3-Methyl adenine-DNA glycosylase (MPG), the initiator protein of BER, thus suggesting that it plays an important role in discriminating the appropriate excision repair system according to the type of DNA lesion. Ncl induces in the nucleolus chromatin de-condensation by binding to histone H1 and plays a role in pre-rRNA transcription and ribosome assembly. As a multifunctional protein, the analysis of Ncl functions are quite challenging due to the broad range of localizations and various corresponding mechanisms. Specific post-translational modifications and its shuttling property further enlarge its pleiotropic functionality, which includes DNA and RNA metabolism, cytokinesis, cell proliferation, angiogenesis, apoptosis regulation, stress response and microRNA processing. Ncl has been increasingly implicated in several pathological processes, especially in carcinogenesis [74] and it has been reported that stress conditions such as heat shock or γ-irradiation could induce a dramatic redistribution of Ncl from the nucleolus to the nucleoplasm in a p53-dependent manner, which may transiently affect DNA replication and repair [75]. Taken together these data indicate that in L areas neoplastic cells tempt to adapt to increased OS by reactivating stress response mechanisms and by recovering key pathways in the maintenance of proteostasis. At the same time the oxidative damage of specific proteins involved in DNA homeostasis and repair extends the persistence of DD providing a molecular basis for neoplastic genomic instability and for the accelerated rate of neoplastic evolution.

At final it is interesting to note that a group of proteins: apolipoprotein A-I (Aap-A1), kinectin (Ktn-1), myosin-9 (Myo-9), spectrin alpha chain (Spec a) and serine/threonine-protein kinase SIK3 (SIK-3) show increased carbonylation levels in PL areas compared to both NPE and L regions suggesting their oxidation characterize the NPE/PL transition while their structural rescue favours the adaptive mechanism observed in cancer.

## 4. Materials and Methods

### 4.1. Patients’ Selection and Enrolment

The local ethical committee fully and preliminarily approved this study (protocol no. CEC/31/16, date 12 January 2016). Patients suffering from chronic solar dermatosis and asking medical advice either to the Regina Elena National Cancer Institute outpatients’ clinic or to the San Gallicano National Dermatological Institute outpatients’ clinic were invited to participate. Inclusion criteria were: (i) to be able to fully understand Italian language; (ii) to have no cognitive impairment; (iii) to have no/have never had neoplastic diseases; (iv) to be not suffering from diabetes mellitus; (v) to have no other dermatological reason to seek for medical advice; (vi) to have not undergone any treatment with biological agents during the past five years. Patients deserving surgical treatment and giving full informed consent were included in the study. Enrolled patients were assisted and treated according to standard criteria of national guidelines and good clinical practice. After the lesion was surgically removed, tissues were fully evaluated by an experienced dermo-pathologist. Provided enough material for an extensive evaluation had collected and forwarded to the histopathological service, from the tissue remnants, pieces of roughly 5 × 5 mm were excised from the central neoplastic area (L) and from the outmost margin of perilesional tissue (PL). An equal amount of non-photo exposed skin (NPE), taken from the remnant of rafts used for plastic reconstructive surgery was used as bona fide non-pathological tissue. Thus, each of the seven cases assayed by redox-proteomic actually consisted in three homologous specimens respectively derived from L, PL and NPE areas.

### 4.2. DNA Extraction and Amplification

DNA extraction was performed with the Zymo Quick-DNA™ Plus Kit (Zymo Research Inc, Irvine CA) used according to the manufacturer indications. Quantity and quality of extracted DNA was evaluated by the A_260_/A_280_ measured in a Nanodrop 2000 spectrophotometer (ThermoFisher Scientific, Waltham, MA, USA). DNA amplification was performed with the SensiFAST SYBR Lo-ROX kit (Bioline GmbH, Luckenwalde, Germany) in a QuantumSudio 6 Flex instrument (Applied BioSystem, Foster City, CA, USA). Cycling conditions were in any case as following: annealing 10′ at 60 °C, extension 6′ at 72 °C, denaturation 10′ at 95 °C for 30 cycles. The p53c72 polymorphism was evaluated by RFLP by *BstUI* digestion as described [19]. Briefly the TP53 exon 4th was amplified with primers P53-72-S (5′-TCC CCC TTG CCG TCC CAA-3′) and P53-72-AS (5′-CAG GGG GAT ACG GCC AGG CA-3′) generating a 339 bp long amplification product. This amplicon was then purified and digested with the BstUI restriction enzyme (New England Biolabs, Ipswich, MA, USA) and the products evaluated through 2.5% Agarose gel electrophoresis visualized under UV-B (312 nm) trans-illumination. The presence of a Guanosine at nucleotide 216, corresponding to an Arginine codon, generates the BstUI specific restriction site (the R72 Arginine variant) whereas the presence of a Cytosine abrogates the BstUI site (the P72 Proline variant). Thus, the presence of two 131 and 208 bp bands stands for the R72 variant whereas the full length 339 bp indicates the P72 variant. Both digested and undigested bands indicate Pro/Arg heterozygous. The allele-specific ARMS-PCR originally developed by Newton et al. [20] was performed according to Sina et al. [21]; the p53 exon 7 sequence was assessed through direct sequencing of PCR specific amplification products by the BigDye Terminator 1.1 Cycle Sequencing Kit (Sanger method). Sequences were aligned to prototype sequence through the BLAST resource at the NCBI (http://www.ncbi.nlm.nih.gov). The presence of Human Papillomavirus genera alpha and beta were detected according to Bauer HH et al. and Berkhout RJ et al. respectively [76,77]. In both cases the conserved L1 region was targeted with a 10ng of starting genomic DNA. In the case of Alpha HPVs, a single amplification was run while in the case of beta HPVs a nested PCR configuration was used as suggested [14]. The amplified products were identified through 2.5% Agarose gel electrophoresys visualized under UV-B (312 nm) trans-illumination. Immunohistochemical detection of alpha and beta HPVs was a courtesy of dr Marisa Gariglio at University of Piemonte Orientale “Amedeo Avogadro”.

### 4.3. Proteins Extraction

Specimens were homogenized using a Wheaton glass homogenizer (~100 passes) in 250 μL of Media I buffer (0.32 M sucrose, 0.10 mM Tris–HCl, pH 8.0, 0.10 mM MgCl_2_, 0.08 mM EDTA, 10 μg/mL leupeptin, 0.5 μg/mL pepstatin, and 11.5 μg/mL aprotinine; pH 8.0). Homogenates were vortexed, aliquoted into Eppendorf tubes, and sonicated with a Fisher 550 Sonic Dismemberer (Pittsburgh, PA, USA) for 10 s at 20% power. Protein concentrations were determined according to the Pierce BCA method (Pierce, Rockford, IL, USA).

### 4.4. Protein Carbonylation

Protein carbonyls are a marker of protein oxidation and were determined as described by Butterfield et al. [78]. Samples (5 μL) were derivatized at room temperature for 20 min in 10 mM DNPH and 5 μL of 12% sodium dodecyl sulphate (SDS). Samples were neutralized with 7.5 μL of neutralization solution (2 M Tris in 30% glycerol). The derived samples (250 ng) were then blotted onto a nitrocellulose membrane under vacuum pressure using a slot-blot apparatus (Bio-Rad, Hercules, CA, USA). Membranes were blocked with 3% bovine serum albumin in Wash Blot, a phosphate-buffered saline (PBS) solution containing 0.04% (*v*/*v*) Tween-20 and 0.10 M NaCl, for 1.5 h and incubated with a 1:100 dilution of rabbit polyclonal anti-DNP primary antibody in Wash Blot for 2 h at room temperature on a rocker. Blots were rinsed three times for 5 min each in Wash Blot and subsequently incubated with a 1:8000 dilution of anti-rabbit IgG alkaline phosphatase secondary antibody in Wash Blot for 1 h at room temperature on a rocker. The membrane was washed three times for 5 min each in Wash Blot and developed using a solution of nitro-tetrazolium blue chloride (0.2 mM) and 5-bromo-4-chloro-3-indolyl phosphate dipotassium (0.4 mM) in ALP buffer (0.1 M Tris, 0.1 M NaCl, 5 mM MgCl_2_; pH 9.5). Dried blots were quantified using Scion Image software (PC version of Macintosh compatible NIH software). Controls, using samples with no primary antibody or samples pre-treated with NaBH_4_ to reduce protein carbonyls, resulted in no staining.

### 4.5. Two-Dimensional (2D) Electrophoresis and Western Blot

For the first-dimension electrophoresis, proteins (200 mg in 200 mL of rehydration buffer) were applied to a ReadyStripTM IPG strip pH 3–10 (Bio-Rad). The strips were soaked in the sample solution for 1 h to allow uptake of the proteins. The strips were then actively rehydrated in Protean IEF Cell Apparatus (Bio-Rad) for 16 h at 50 V. The isoelectric focusing was performed at 300 V for 2 h linearly; 500 V for 2 h linearly; 1000 V for 2 h linearly, 8000 V for 8 h linearly and 8000 V for 10 h rapidly. All the processes above were carried out at room temperature. The focused IEF strips were stored at −80 °C until second dimension electrophoresis was performed. For second dimension electrophoresis, thawed strips were equilibrated for 10 min in 50 mM Tris-HCl (pH 6.8) containing 6 M urea, 1% (*w*/*v*) SDS, 30% (*v*/*v)* glycerol, and 0.5% dithiothreitol, and then re-equilibrated for 15 min in the same buffer containing 4.5% iodacetamide in place of dithiothreitol. 12% Precast criterion gels (Bio-Rad) were used to perform second dimension electrophoresis. Precision ProteinTM Standards (Bio-Rad) were run along with the sample at 200 V for 65 min. After electrophoresis, the gels were fixed (7% acetic acid, 10% methanol) and stained with Bio-Safe Coomassie Gel Stain (Bio-Rad). To identify carbonylated proteins, samples (200 mg proteins) were derivatized as above described, subjected to 2-DE and transferred to nitrocellulose membrane using Criterion Blotter apparatus (Bio-Rad) at 100 V for 1 h. The carbonylated proteins were detected as above reported.

### 4.6. Image Analysis

The 21 gels (*n* = 7 NPE controls, *n* = 7 PL and *n* =7 L) and corresponding nitrocellulose blots images were obtained using a Chemidoc MP System (Bio-Rad). All the images were saved in TIFF format. 2D gels and 2D blots images (8 gels and 8 blots for each group of study) were analysed by PD-Quest 2D Analysis (7.2.0 version; Bio-Rad). PD-Quest spot-detection software allows the comparison of 2D gels as well as 2D blots, from NPE, PL and L groups. Briefly, a master gel was selected followed by normalization of all gels and blots according to the total spot density. Gel-to-blot analysis was then initiated in two parts. First, manual matching of common spots was performed, that could be visualized among the differential 2D gels and 2D blots. After obtaining a significant number of spots, the automated matching of all spots was then initiated. Automated matching is based on user-defined parameters for spot detection. These parameters are based on the faintest spot, the largest spot, and the largest spot cluster that occur in the master gel and are defined by the user. This process generates a large pool of data, approximately 400–800 spots. Only proteins showing computer-determined significant differential levels between the groups being analysed were considered for identification. To determine significant differential levels of proteins, analysis sets were created using the analysis set manager software incorporated into the PD-Quest software. The numbers of pixels that occur in a protein spot were computed by the software corresponding to an increase/decrease in protein level. The image analysis was conducted first on blot and then on Sypro Ruby-stained expression gels. The two analyses were compared by software to normalize HNE-modified protein value to expression value for each spot matched.

### 4.7. In-gel Trypsin Digestion/Peptide Extraction

Protein spots identifies as significantly altered from our group comparison were excised from 2D-gels and transferred to individual Eppendorf microcentrifuge tubes for trypsin digestion as described previously [79]. In brief, DTT and IA were used to break, and cap disulphide bonds and the gel plug was incubated overnight at 37 °C with shaking in modified trypsin solution. Tryptic peptide solutions were reconstituted in water and stored at −80 °C until MS/MS analysis.

### 4.8. RP-HPLC-High Resolution MS/MS Characterization of Tryptic Peptides

High-resolution HPLC-ESI-MS/MS experiments were carried out by an Ultimate 3000 RSLC nano system coupled to an LTQ Orbitrap ELITE apparatus (Thermo Fisher Scientific, Waltham, MA, USA). Zorbax 300 SB-C18 (3.5 μm particle diameter; column dimension 1 mm × 150 mm) (Agilent Technologies, Santa Clara, CA, USA) was used as chromato-graphic column. The following eluents were used: (A) 0.1% (*v*/*v*) aqueous FA and (B) 0.1% (*v*/*v*) FA in ACN/water 80/20 *v*/*v*. The ap- plied gradient was: 0–2 min 5% B, 2–40 min from 5 to 70% B (linear), 40–45 min from 70 to 99% B (linear), at a flow rate of 50 μL/min with a total run of 65 min. MS spectra were collected with 120,000 resolution and m/z range from 350 to 2000. In data-dependent acquisition mode the five most intense multiply charged ions were selected and fragmented in ion trap by using CID 35% normalized collision energy. Tuning parameters were: capillary temperature 300 °C, source voltage 4.0 kV.

### 4.9. Chemicals and Biochemicals

All other materials used, unless otherwise specified, were analytical grade products purchased from the current laboratory suppliers either Sigma–Aldrich (St. Louis, MO, USA) or Bio-Rad (Bio-Rad Laboratories, Milan, Italy).

### 4.10. Statistical Analysis

Raw data for BD PAGE and PD-Quest image analyses are available online at the link: https://gbox.garr.it/garrbox/index.php/apps/files/?dir=/&fileid=27647908.

Two-sided, Student’s *t*-tests were used to analyse differences in protein levels between PL and L areas. A *p*-value of less than 0.05 was considered statistically significant. The significance of the change in carbonylation of specific proteins in the proteomics study was evaluated via non-parametric Mann–Whitney–Wilcoxon test. *p* < 0.05 was considered statistically significant.

## 5. Conclusions

In perilesional areas, i.e., chronically sun over-exposed lesions, the incoming UV radiation, beside its primary genotoxic activity, specifically impairs the DNA and proteome repairing mechanisms, suppresses the proteasomal activity and disrupts apoptosis signalling. As a consequence, DNA damage and proteome misfunction tend to persist increasing the chance that damaged or inappropriately repaired DNA undergoes replication. Pro-carcinogenic mutations are, therefore, generated at an increased pace and potentially carcinogenic cells, inefficiently cleared by the concomitantly dysregulated apoptosis can establish and grow. The cell/matrix signalling, inflammation and immune response are also critically subverted, unleashing transformant clones to expand and progress to a full neoplastic phenotype.

In neoplastic areas, in agreement with literature data, a comparatively lower oxidative burden is found as a possible result of a pro-survival adaptive response. In this context, proteins involved in DNA homeostasis and repair are selectively damaged, providing a basis for an accelerated neoplastic evolution. As a whole, the UV component of solar radiation, in addition to be a cancer initiating agent, acts as a cancer promoting agent and concur to the neoplastic progression of established lesions.

These data, once confirmed and extended by further studies to come, disclose a few translational implications. Indeed, the prevalence of conditions of the actinic keratosis/squamous cell carcinoma spectrum are steadily growing at any latitude all over the world and their diagnosis and prognostic evaluation, still largely based on purely morphological criteria, remains unsatisfactory. Thus, protein oxidation as such, or a combination of related molecular adducts, might serve as a basis for the development of innovative protocols for their diagnosis and their staging. In terms of preventive medicine, the avoidance of protein oxidation and/or its possible correction through exogenous supply should be included among the criteria for designing and authorising new sunscreen products.

## Figures and Tables

**Table 1:** Synopsis of patients’ clinical presentation, histopathological diagnoses, p53 and HPV status.

N	Sex	Age (years)	Clinical Presentation	Istological Diagnosis	P53 Codon 72 Polymorphism	HPV Status		
						Alpha HPVs	Beta HPVs	E4 Protein Expression
1	M	62	AK	AK	A/A	ND	ND	ND
2	M	78	AK	AK	P/A	ND	ND	ND
3	M	69	AK	AK	P/P	ND	ND	ND
4	F	68	Forehead neoformation	SCC	P/A	ND	ND	ND
5	M	71	Ear pavillon neoformation	SCC	P/A	ND	ND	ND
6	M	60	Scalp Skin neoformation	Bowen’s D.	P/A	ND	ND	ND
7	M	82	Left cheek neoformation	BCC	P/A	ND	ND	ND
8	F	82	Left cheek neoformation	BCC	P/A	ND	ND	ND
9	F	71	Nose skin neoformation	BCC	P/A	ND	ND	ND
10	F	93	Left cheek neoformation	SCC	A/A	Positive	ND	ND
11	F	72	Left cheek neoformation	BCC	A/A	Positive	Positive	ND
12	M	75	Ear pavillon neoformation	SCC	P/A	ND	ND	ND
13	M	79	Scalp Skin neoformation	AK	A/A	ND	ND	ND
14	F	63	Left cheek neoformation	BCC	P/A	ND	ND	ND
15	M	74	AK	BCC	A/A	ND	ND	ND
16	M	87	AK	BCC	P/A	ND	ND	ND
17	M	80	AK	AK	A/A	ND	ND	ND

**Table 2:** Data from proteomics and mass spectrometry (MS) analysis. Each protein identified is characterized by the spot number on gel, the Uniprot ID number, the gene acronym, the identification score, the peptides coverage, the putative molecular weight (MW), the calculated isoelectric point (pI) and the fold of oxidation in the group of comparison (PL vs. NPE or PL vs. L).

Spot N.	Uniprot ID	PROTEIN	GENE	Score	Coverage	MW [kDa]	Calc. pI	Fold of Oxidation	
								PL/NPE	PL/L
401	P07237	Protein disulfide-isomerase A1 (PDIA1)	*P4HB*	8.09	5.91	57.1	4.87	11.65	ns
	P08238	Heat shock protein HSP 90 (HSP90)]	*HSP90AB1*	8.81	5.94	83.2	5.03		
506	P27816	Microtubule-associated protein 4 (MAP-4)	*MAP4*	6.22	2.17	120.9	5.43	5.15	ns
	P54652	Heat shock-related 70 kDa protein 2(HSP72)	*HSPA2*	6.35	3.29	70.0	5.74		
601	P27797	Calreticulin (Calr)	*CALR*	21.23	16.55	48.1	4.44	68.82	ns
602	P14625	Endoplasmin (GRP94)	*HSP90B1*	23.52	11.58	92.4	4.84	39.96	ns
	Q07507	Dermatopontin (Dpt)	*DPT*	16.85	17.91	24.0	4.82		
1003	P07951	Tropomyosin beta chain (Tpm-2)	*TPM2*	15.60	19.01	32.8	4.70	81.3	ns
	P08758	Annexin A5 (Anx-5)	*ANXA5*	14.23	10.00	35.9	5.05		
1207	P50502	Hsc70-interacting protein (Hip)	*ST13*	7.87	8.40	41.3	5.27	0.2	ns
2302	P54727	UV excision repair protein RAD23B (HR23B)	*RAD23B*	13.40	14.91	43.1	4.84	ns	0.04
2803	P19338	Nucleolin (Ncl)	*NCL*	15.24	4.79	76.6	4.70	ns	0.14
3101	P31947	14-3-3 protein sigma (14-3-3 σЦ)	*SFN*	54.21	22.98	27.8	4.74	17.57	ns
	P55327	Tumor protein D52 (Tpd-52)	*TPD52*	13.43	16.52	24.3	4.83		
	Q15293	Reticulocalbin-1 (Rcn1)	*RCN1*	8.81	7.85	38.9	5.00		
	P28072	Proteasome subunit beta type-6 (Psmb-6)	*PSMB6*	8.81	12.97	25.3	4.92		
3202	P02647	Apolipoprotein A-I (Apo-A1)	*APOA1*	99.74	43.07	30.8	5.76	93.26	12.5
3601	P55072	Transitional endoplasmic reticulum ATPase (TER ATPase)	*VCP*	10.88	7.07	89.3	5.26	ns	25
3702	P45974	Ubiquitin carboxyl-terminal hydrolase 5 (UCH-5)	*USP5*	17.06	10.02	95.7	5.03	21.74	
4801	Q86UP2	Kinectin (Ktn-1)	*KTN1*	19.56	7.00	156.2	5.64	0.04	0.24
	P35579	Myosin-9 (Myo-9)	*MYH9*	90.91	13.11	226.4	5.60		
	Q13813	Spectrin alpha chain, non-erythrocytic 1 (Spec)	*SPTAN1*	16.42	3.16	284.4	5.35		
5103	O43399	Tumor protein D54 (Tpd-52l2)	*TPD52L2*	12.85	18.93	22.2	5.36	6.92	ns
	Q06323	Proteasome activator complex 1 (Pac-1)	*PSME1*	9.24	28.51	28.7	6.02		
6101	P32119	Peroxiredoxin-2 (Prdx-2)	*PRDX2*	109.53	40.91	21.9	5.97	ns	7.7
	Q99497	Protein/nucleic acid deglycase DJ-1 (DJ-1)	*PARK7*	18.86	23.81	19.9	6.79		
	Q9ULZ3	Apoptosis-associated speck-like protein containing a CARD (hASC)	*PYCARD*	10.83	19.49	21.6	6.34		
6404	Q6S8J3	POTE ankyrin domain family member E (POTE-2)	*POTEE*	30.89	7.91	121.3	6.20	ns	11.1
6602	Q9Y2K2	Serine/threonine-protein kinase SIK3 (SIK-3)	*SIK3*	6.61	2.14	139.9	6.70	6.43	8.3
7501	P01023	Alpha-2-macroglobulin (Alpha-2-M)	*A2M*	14.77	6.04	163.2	6.46	ns	20.7
7803	Q9H0J4	Glutamine-rich protein 2 (Qrich-2)	*QRICH2*	1.72	2.71	180.7	6.73	ns	20.5
5602	P18206	Vinculin (Vcl)	*VCL*	32.83	9.26	123.7	5.66	5.62	ns
	P02452	Collagen alpha-1(I) chain (Alpha-1 t-I col)	*COL1A1*	8.32	1.84	138.9	5.80		

**Table 3:** Function and putative role in ultraviolet B (UVB) damage and cancer progression of the proteins identified differentially carbonylated. The table reports only the proteins mainly involved in the carcinogenesis process.

Protein	Oxidation	Function	Putative Role in UVB Damage and Cancer Progression
PDI-A1	↑ PL vs. NPE	Chaperoning and stress response; protein folding/degradation	Reduced induction of stress response and protein quality control pathways in PL areas
HSP-90	↑ PL vs. NPE		
HSPA2	↑ PL vs. NPE		
Psmb6	↑ PL vs. NPE		
Calr	↑ PL vs. NPE		
GRP94	↑ PL vs. NPE		
TER ATPase	↑ PL vs. L	Chaperoning and stress response; protein folding/degradation	Increased induction of stress response and protein quality control pathways in L areas
Prdx-2	↑ PL vs. L		
DJ-1	↑ PL vs. L		
Psmb6	↑ PL vs. L		
Anx-5	↑ PL vs. NPE	Apoptosis/survival	Reduced activation of apoptotic response in PL areas
UCH-5	↑ PL vs. NPE		
Vcl	↑ PL vs. NPE	Cell architecture, adhesion and motility	Alteration of cell structure and functionality in PL areas
Dpt	↑ PL vs. NPE		
Apo-AI	↑ PL vs. NPE		
HR23B	↓ PL vs. L	DNA damage repair	Increased DNA damage and genome instability in L areas
Ncl	↓ PL vs. L		

## Abbreviations

**Acronym**	**Extended Name**
6-4 PP	Pyrimidine (6-4) pyrimidone photoproducts
Aap-A1	Apolipoprotein A1
Anx-5	Annexin-5
ARMS-PCR	Amplification Refractory Mutation System-PCR
CPD	Cyclobutane Pirimidine Dimer
Calr	Calreticulin
DD	DNA Damage
DDR	DNA Damage Repair
DJ-1	Protein/nucleic acid deglycase DJ-1
DNPH	Formaldehyde-2,4-dinitrophenylhydrazone
Dpt	Dermatopontin
ECM	extracellular matrix
tER	Transitional Endoplasmic Reticulum
GG-NER	Global Genomic-NER
GRP-94	94 kDa glucose-regulated protein (Endoplasmin)
HPV	Human Papillomavirus
HSPA2	Heat Shock-related 70 kDa Protein 2
HSP90B	Heat shock protein HSP 90-beta
L	Lesional area
MHC	Major Histocompatibility Complex
MPG	3 Methyladenine-DNA Glycosylase
Ncl	Nucleolin
NER	Nucleotide Excision Repair
NMSC	Non-melanoma skin cancer
NPE	Non photoexposed area
OS	Oxidative Stress
PDI-A1	Protein Disulfide Isomerase A
PL	Perilesional area
PLC	Protein Loading Complex
Prx-2	Peroxiredoxin-2
PQC	Protein Quality Control
Psmb6	Proteasome subunit beta type-6
RFLP	Restriction Fragment Length Polymorphism
RAD23B	UV excision repair protein RAD23 homolog B
ROS	Reactive Oxygen Species
TGFb1	Tumour Growth Factor beta 1
TP53BP1	Tumour suppressor p53-binding protein 1
UCH5	Ubiquitin carboxyl-terminal hydrolase 5
UPR	Unfolded Proteins Response
UVR	UV Radiation
Vcl	Vinculin
Vop	Transitional endoplasmic reticulum ATPase
XP	Xeroderma Pigmentosum
XP(A-C)	Xeroderma Pigmentosum complementation group (AC)
